# Time-bin entanglement at telecom wavelengths from a hybrid photonic integrated circuit

**DOI:** 10.1038/s41598-024-60758-4

**Published:** 2024-05-01

**Authors:** Hannah Thiel, Lennart Jehle, Robert J. Chapman, Stefan Frick, Hauke Conradi, Moritz Kleinert, Holger Suchomel, Martin Kamp, Sven Höfling, Christian Schneider, Norbert Keil, Gregor Weihs

**Affiliations:** 1 Institut für Experimentalphysik, Universität Innsbruck, 6020 Innsbruck, Austria; 2 Faculty of Physics and Vienna Doctoral School in Physics and Vienna Center for Quantum Science and Technology, University of Vienna, 1090 Vienna, Austria; 3 Fraunhofer Institute for Telecommunications, Heinrich-Hertz-Institut, 10587 Berlin, Germany; 4 Department of Physics, Optical Nanomaterial Group, Institute for Quantum Electronics, ETH Zurich, 8093 Zurich, Switzerland; 5 Technische Physik, Universität Würzburg, 97074 Würzburg, Germany; 6 Institute of Physics, University of Oldenburg, 26129 Oldenburg, Germany

**Keywords:** Materials for optics, Nonlinear optics, Quantum optics, Quantum information, Photonic devices

## Abstract

Mass-deployable implementations for quantum communication require compact, reliable, and low-cost hardware solutions for photon generation, control and analysis. We present a fiber-pigtailed hybrid photonic circuit comprising nonlinear waveguides for photon-pair generation and a polymer interposer reaching ${68}\,\hbox {dB}$ of pump suppression and photon separation based on a polarizing beam splitter with $>{25}\,\hbox {dB}$ polarization extinction ratio. The optical stability of the hybrid assembly enhances the quality of the entanglement, and the efficient background suppression and photon routing further reduce accidental coincidences. We thus achieve a $\left( 96_{-8}^{+3}\right) \%$ concurrence and a $\left( 96_{-5}^{+2}\right) \%$ fidelity to a Bell state. The generated telecom-wavelength, time-bin entangled photon pairs are ideally suited for distributing Bell pairs over fiber networks with low dispersion.

## Introduction

As data traffic continues to grow, the cryptography community is increasingly aware of the importance of methods and devices ensuring an efficient and secure data transmission. For the required security, the conventional public key infrastructure has been shown to be unsuitable in the long term^1–3^. Quantum communication, in contrast, provides information-theoretical security when implemented correctly^4^. A multitude of implementations have been demonstrated in field trials using metro networks^5–7^. Among those, the majority do not rely on entanglement and the experimental setups have the size of a computer rack or larger. For mass-deployment and practical implementation, however, quantum communication systems must become more compact, cost-effective and scalable. This can be achieved via quantum system-on-chip modules^8–10^. Also allowing for individual optimization of dissimilar components, hybrid photonic integrated circuits (PICs) have recently received much attention in quantum photonics^11,12^, where challenges ranging from single-photon generation to reconfigurable photon routing and high-efficiency detection have particularly demanding requirements that cannot be fulfilled by a single photonic platform.

In addition to scaling up quantum communication systems, one must strive for more than conditional security. It will be difficult to certify all quantum communication source and receiver modules and to ensure their long-term integrity. Therefore, entanglement-based quantum key distribution (QKD) schemes are promising, especially when used in future device-independent schemes that rely on the principle of non-locality and can generate secure keys even for untrusted devices^13–16^.

For pratical QKD, the transmitted qubits must be compatible with the existing telecom infracture and also conserve the entanglement en route. To this end, time-bin entanglement is especially well suited as it does not suffer from decoherence due to polarization mode dispersion^17,18^. In this scheme, a photon pair is created in a coherent superposition of two time bins and the communicating parties each receive one of the photons allowing them to test the quality of the entanglement and generate bits of a shared secret key. A number of experiments have demonstrated this form of entanglement as a proof-of-principle for entanglement-based QKD^19^, using integration-ready sources^20–22^, generating on-demand time-bin qubits^23^, or achieving a distance record^24^. However, few telecom time-bin entanglement sources have been realized on-chip or in optical fiber^25–27^.

We present in this article an on-chip, partially fiber-pigtailed source of time-bin entangled photon pairs in the telecom wavelength range working at room temperature. The photon pairs are generated in a nonlinear crystal made of aluminum gallium arsenide, called a Bragg-reflection waveguide (BRW)^28–30^. This source is integrated with a polymer chip, the PolyBoard, which hosts all passive optical components including a long-pass filter (LP) showing ${68}\,\hbox {dB}$ of pump suppression, a polarizing beam splitter (PBS) achieving $>\,$25 dB polarization extinction ratio $\left( P\!E\!R\right)$, and specially designed grooves for fiber pigtailing^31–33^. We achieve a coincidence rate of 460 Hz per mW continuous-wave (CW) external pump power between the signal and idler photons without correcting for fiber loss or detector efficiency. In the time-bin entanglement scheme, this results in photon pair rates of 1.4 Hz per mW of external pump power, a concurrence of $\left( 96_{-8}^{+3}\right) \%$ and a fidelity of $\left( 96_{-5}^{+2}\right) \%$ to the $\mathinner {|{\Phi ^+}\rangle }$ Bell state. By using a hybrid integration approach, we benefit from the strong nonlinearity of aluminum gallium arsenide to generate photon pairs and the linear optical capabilities of the PolyBoard technology. Our approach can be applied to other photonics platforms to gain maximum benefit from different technologies.

The article is structured as follows: After a brief explanation of the time-bin scheme, both the BRW and the PolyBoard are introduced in more detail followed by a section on their hybrid integration and assembly process. We then perform a classical characterisation of the PIC and finally present the time-bin measurements including state tomography using maximum likelihood estimation^34,35^.

## Materials and methods

**Figure 1:**
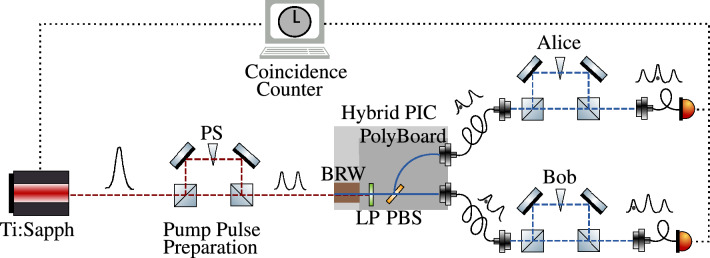
Time-bin entanglement scheme. Our setup includes the preparation of pump pulse pairs, the photonic integrated circuit (PIC), where the telecom photon pairs are created in the Bragg-reflection waveguide (BRW), filtered at a long-pass filter (LP), separated at a polarizing beam splitter (PBS) and coupled into fiber, as well as the stations of Alice and Bob, where the entanglement is analyzed. These consist of interferometers with variable phase shift (PS) and single photon detectors.

We implement the time-bin entanglement as illustrated in Fig. 1. A coherent superposition of time bins is prepared by passing a (transform-limited) pulsed Ti:Sapphire laser (Coherent Mira 900) with 76 MHz repetition rate and 0.8 nm full width at half maximum (FWHM) bandwidth emitting at 767 nm through an asymmetric free-space Michelson interferometer. This splits each pulse into an early and a late time bin separated by a 3 ns delay and the pulse pair then travels to the hybrid PIC. One photon pair is produced with probability $p\ll 1$ by either the early or late pump pulse, separated by polarization and routed to two optical fibers on the hybrid PIC. The photons are sent to two parties, Alice and Bob, who analyze the entanglement via interferometers with the same delay as the pump interferometer. In our setup, all three interferometers are folded into the same physical interferometer, as shown in the supplementary document in Section 3. Finally, the photons are measured by superconducting-nanowire-single-photon detectors (SNSPDs) (Single Quantum Eos 720 CS, Au Line) with 40 ps timing jitter and $>\,$60 % detection efficiency. A triple coincidence between the pump pulse and the photons detected by Alice and Bob is computed via a time tagger (Swabian Time Tagger Ultra) with 10 ps rms jitter and 2 ns dead time.

**Figure 2:**
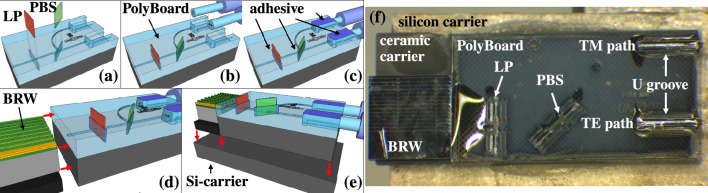
Assembly process and photograph of the hybrid PIC. First, the PolyBoard is prepared by inserting the thin-film long-pass filter (LP) and polarizing beam splitter (PBS) in their pre-etched slots (**a**), installing the output fibers (**b**), optimizing all elements for transmission and securing them with UV-curing, index-matched adhesive (**c**). Next, using active alignment, the BRW is end-facet coupled to the PolyBoard and the interface secured with adhesive once the transmission is optimized (**d**). Finally, the newly formed hybrid PIC is mechanically stabilized by a common silicon mount with 7*x*10 mm footprint (e). A photograph (reprinted with permission from^50^) of the final assembly used in this work (**f**).

The hybrid PIC comprises a nonlinear BRW with a high $\chi ^{\left( 2\right) }$ nonlinear coefficient enabling efficient parametric down-conversion (PDC)^36,37^, and the PolyBoard, a passive optical interposer. The assembly process and final chip are shown in Fig. 2.

To provide waveguiding and modal phase-matching, the BRW is made up of layers with different aluminum concentrations and etched into a ridge structure. By carefully engineering the layer thicknesses and aluminum concentrations^38^, and by reducing the waveguide ridge sidewall roughness^39^ we achieve photon-pair rates of up to $(8.9 \pm 0.5) \cdot 10^{4}$ Hz per mW of external pump power, which corresponds to about $4 \cdot 10^{5}$ Hz per mW of internal pump power and $4 \cdot 10^{3}$ per mW of internal pump power and nm bandwidth^40^. These count rates are directly measured at the detectors and not corrected for transmission and coupling losses or detector efficiencies. The photon pairs generated in the telecom wavelength range benefit from minimal signal attenuation in the existing fiber infrastructure. Because of their broad-band ($\sim$100 nm) emission, BRWs can also be considered for the distribution of entanglement in multiple telecom channels. In addition to being correlated in their time of creation, which is used for time-bin entanglement, the two photons of a pair are anti-correlated in wavelength and polarization, opening up the possibility for other forms of entanglement or even hyperentanglement^41,42^. Achievements realized with BRWs thus far include the generation of polarization entanglement^43–45^, energy-time entanglement^46^, and free-space time-bin entanglement^22^, as well as the integration of an internal pump laser^47,48^ and with it the demonstration of difference-frequency generation^49^.

The photons generated by PDC in the BRW are orthogonally polarized and collinear with the pump light. It is therefore necessary to spectrally filter the pump and convenient to separate the photons with a polarizing beamsplitter. The required components are technologically challenging to realize and therefore we employ a hybrid integration with polymer waveguide circuits^31^.

Polymer-based PICs feature lower production and material cost than standard semiconductor platforms^51,52^, a large transparency window, and an effective index that closely matches silica fibers allowing low-loss pigtailing. The presented interposer features two custom-made, dielectric thin-film elements, a LP to reject the pump light and PBS redirecting orthogonal polarizations to separate waveguides. The input waveguide facet is diced for end-facet coupling to the BRW, whereas the output waveguides are directly pigtailed with standard polarization maintaining (PM) fibers in a U-groove arrangement^31^, which improves the mechanical stability.

Hybrid integration, as employed between the BRW and the PolyBoard in this work, combines the strengths of both material platforms and can also introduce new features missing in the monolithic counterparts. The PolyBoard has proven its versatility by implementing on-chip free space sections^32^, thermal phase shifters or switches^33^, tunable distributed Bragg-reflector lasers^53,54^, on-chip isolators and circulators^55,56^, and various integrated circuits for quantum photonics^50^.

When interfacing dissimilar platforms, the mode field overlap is crucial for the coupling loss. The complicated layer structure of the BRW gives rise to a non-rotationally symmetric mode with a shape resembling two stacked oblong ovals. Thus, the current design results in a mode field overlap with the near-Gaussian mode of the PolyBoard of $\sim$55% (for more details see the supplementary document Sections 1 and 2) but mode-engineering via taper structures can boost the overlap significantly ^57,58^. For instance, a tapered section in the BRW could help make the modes there less elongated in the horizontal direction. The assembly of the hybrid PIC is a multi-step process with active alignment using the telecom laser transmission signal and is sketched in Fig. 2a–e.

## Results

We perform a series of classical characterization measurements using a CW laser to evaluate the performance of the individual components of the PIC. The results provide insights in addition to the coincidence measurements at the few-photon level and, furthermore, are less sensitive to noise.

**Figure 3:**
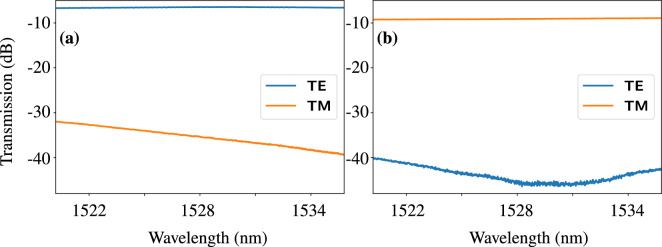
Transmission measurements of the PolyBoard interposer. The input polarization is set to transversal-electric (TE) and transversal-magnetic (TM) and the transmission is measured at both output paths while the laser wavelength is scanned. The polarizing beam splitter (PBS) predominantly transmits TE-polarized light and reflects TM-polarized light. For both the TE path (**a**) and the TM path (**b**), the polarization extinction ratio is calculated as the difference between TE and TM transmission. The spectral dependence of the suppressed polarization is ascribed to chromatic effects in the PBS thin-film layer stack.

To this end, we couple a CW laser into the diced facet of the PolyBoard and measure the transmission of both output fibers for transversal-electrically (TE) and transversal-magnetically (TM) polarized input light while scanning the laser wavelength (see Fig. 3). For both outputs, we find a flat transmission curve for the favored polarization with an average loss of $(6.54 \pm 0.08)$ dB for the TE and $(9.1 \pm 0.1)$ dB for the TM path. Note that this measurement also includes the input coupling loss of 0.5 dB to 1 dB. From test structures featuring only a single optical element, we estimate $\sim {1.5}\,\hbox {dB}$ coupling loss per U-groove, $\sim {0.8}\,\hbox {dB}$ propagation loss and $\sim {1}\,\hbox {dB}$ excess loss of each of the TFEs. However, the filter slots for this PIC are slightly wider than targeted due to inaccuracies during the etching and are expected to cause slightly higher loss. This also results in a slight out-of-plane deflection from the inserted PBS causing the lower transmission in the TM path. Further, we infer the polarization extinction ratios by comparing the transmission measurements for orthogonal polarizations, (as shown in Fig. 3), yielding $P\!E\!R>{30}\,\hbox {dB}$ for the reflection and $P\!E\!R>{25}\,\hbox {dB}$ for the transmission port of the PBS.

The suppression of pump light by the LP cannot be measured directly at the PolyBoard but is estimated from a separate test structure. Using a white light source, we find a suppression exceeding 40 dB for the range of 700–850 nm limited only by the noise floor of our detector. Employing a laser diode emitting at 785 nm, we verify a suppression of $(68\pm 1)$ dB, while the loss of the LP at telecom wavelengths amounts to $\sim {0.9}\,\hbox {dB}$ (for more details see the supplementary document Section 1).

We conclude that the PolyBoard not only reduces size and cost of the implementation drastically but also provides high-performance polarization splitting and long-pass filtering that easily matches or even outperforms bulk elements albeit at lower transmission.

Moving on to characterizing the quantum performance of our PIC, we pump PDC by coupling 767 nm CW light (M2 SolsTiS) into the BRW input facet. We employ a CW laser here for easier comparison of the performance with (previous) bare BRW samples. We measure a coincidence rate of 460 Hz per mW external pump power between the signal and idler photons without correcting for fiber loss or detector efficiency. The coincidence rate is consistent with this BRW’s stand-alone performance considering the losses expected from hybrid integration on the PIC described above.

For the time-bin entanglement measurement and state tomography, we follow the methods described by James et al.^34^ and Takesue et al.^35^. Our time-bin entanglement scheme shown in Fig. 1 produces photons in two bases: the time basis made up of the early ($\mathinner {|{1}\rangle }$) and the late ($\mathinner {|{2}\rangle }$) time bins and the energy basis made up of superpositions of $\mathinner {|{1}\rangle }$ and $\mathinner {|{2}\rangle }$ manifesting in the central time bin. Both bases are measured simultaneously by detecting the arrival times of photons sent to Alice and Bob. For all of the following we use the pulsed laser, an external pump power of 1 mW, a 200 ps bin width, and 360 s integration time. The accidental coincidences rate is $(1.5 \pm 0.2) \cdot 10^{-3}$ Hz. We reference the arrival times of photons at Alice’s and Bob’s detectors to a trigger given by a photodiode installed in the path of the pulsed pump laser. Due to the limited transmissions of the free-space interferometers of 5 % to 7 %, we measure a total coincidence count of about 1.4 Hz per mW external pump power. Correcting for the loss in the two telecom interferometers we obtain a coincidence rate of 290 Hz to 560 Hz. By rotating the phase plate in one of the interferomters, we reveal the interference in the central time bin with a $(91\pm 5)$ % visibility.

**Figure 4:**
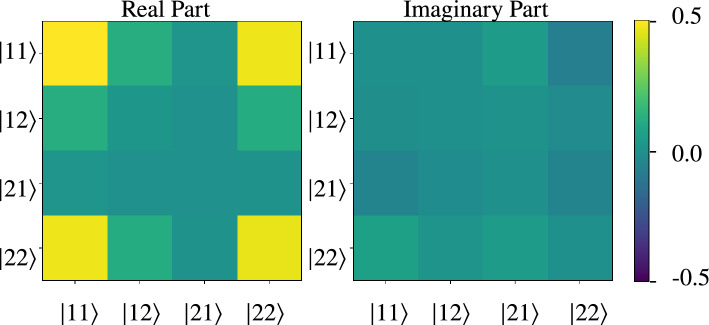
**Density matrix reconstructed via maximum likelihood estimation.** The real and imaginary parts of the density matrix demonstrate the high degree of entanglement and fidelity to the $\mathinner {|{\Phi ^+}\rangle }$ Bell state.

From the triple coincidence between the trigger and Alice’s and Bob’s detectors results a 2D histogram, an example of which is shown in the supplementary document in Section 4 in Fig. S5. We perform a measurement for four phase plate settings in Alice’s and Bob’s interferometers corresponding to the states $\mathinner {|{++}\rangle }$, $\mathinner {|{+L}\rangle }$, $\mathinner {|{L+}\rangle }$, and $\mathinner {|{LL}\rangle }$, where $\mathinner {|{+}\rangle }=1/\sqrt{2}\left( \mathinner {|{1}\rangle }+\mathinner {|{2}\rangle }\right)$ and $\mathinner {|{L}\rangle }=1/\sqrt{2}\left( \mathinner {|{1}\rangle }+i\mathinner {|{2}\rangle }\right)$. These are two-photon states where both photons were measured in the energy basis, meaning the central time bin. From these, we obtain the coincidence counts (without correcting for accidentals) for projections onto 16 different two-photon states serving as input for the state tomography. This method is explained in detail in Ref.^35^ and illustrated in the tables included in that publication. As the linear reconstruction of the density matrix leads to negative eigenvalues and therefore an unphysical state, we employ a maximum likelihood estimation to recover the density matrix shown in Fig. 4 (values can be found in the supplementary document in Section 5). From this we obtain a concurrence of $\left( 96_{-8}^{+3}\right) \%$, a $\left( 96_{-5}^{+2}\right) \%$ fidelity to the $\mathinner {|{\Phi ^+}\rangle }$ Bell state and an expected Bell S-parameter of $\left( 2.70_{-0.33}^{+0.09}\right) \%$. The uncertainties are derived using a Monte Carlo simulation where we create $10^4$ sets of coincidence counts with Poissonian distribution around the actually measured counts and perform the maximum likelihood estimation for each. The results demonstrate both strong entanglement and the possibility to violate the Clauser-Horne-Shimony-Holt (CHSH) Bell inequality with the generated pairs. The resulting nonlocal correlations are a useful resource for quantum communication tasks.

## Discussion and conclusion

The mass-deployment of entanglement-based QKD transceivers requires a high level of integration while components must comply with the challenging operation in a real-life environment based on noisy, dispersive fiber networks. Despite the plethora of miniaturized quantum-light sources, only a few monolithic solutions exist that enable additional functionalities such as photon filtering or splitting on-chip making hybrid integration worthwhile^11,12^. Here, we present a particularly versatile solution combining a nonlinear BRW with the polymer-based PolyBoard for light filtering and routing that is easily adaptable to other wavelengths, filter or encoding (e.g polarization) requirements^50^. The PIC was developed as part of the EU Quantum Flagship project Uniqorn which explored different material platforms for quantum technologies. While hybrid integration in general is a promising way forward, the PolyBoard platform especially has proven its versatility as a host for quantum light sources, single-photon-avalanche detectors, and quantum random number generators^50^.

The pair emission rate of our PIC is consistent with previous experiments using BRWs in our group^22^ and comparable to those of others^30^. Further enhancement of the coincidence rates involves optimizing the design of the hybrid PIC. First, engineering the BRW’s and the PolyBoard’s mode field at the intersection using tapered waveguides reduces loss due to mode mismatch ^57,58^. Second, employing the latest generation of BRWs–featuring photon-pair generation rates >60 times higher than the sample used here^40^–may decrease the needed pump power and relax the requirements for low loss down the line. In the current implementation, we identify the free-space interferometers as the dominating source of loss and therefore as the bottleneck on our way to efficiently produce entangled photon pairs. Actively stabilizing the interferometers can improve the spatial overlap of beams as well as the temporal overlap of pulses and counteract some of the degradation in the classical visibilities^59^. However, the free-space interferometers are not at the core of this work. Exchanging them for chip- or fiber-based counterparts may not only improve the efficiency but also promotes miniaturization further.

In contrast to the modest coincidence rates, the demonstrated entanglement is very strong with a concurrence of $\left( 96_{-8}^{+3}\right) \%$ and a fidelity of $\left( 96_{-5}^{+2}\right) \%$ to a Bell state. Lower uncertainties can be achieved with higher count rates once the interferometers have been replaced. Already now, our PIC compares well with other telecom time-bin entanglement demonstrations, including the $(88.9 \pm 1.8)$ % concurrence and $(94.2 \pm 0.9)$ % fidelity measured for a bare BRW^22^ in free space, the $(74.1 \pm 4.8)$ %^25^ and 91 %^60^ coincidence fringe visibilities found for fiber-based approaches, the 92 % visibility measured in a CW time-bin experiment^61^, and the $(91.0 \pm 0.7)$ % fidelity quoted for an all on-chip implementation^27^.

We attribute the increased purity of the entanglement to the hybrid integration of BRW and PolyBoard, as the PIC offers optical stability and the end-facet coupling reduces the amount of unwanted photoluminescence picked up from the BRW^62^. Moreover, the $P\!E\!R$ of $>{25}\,\hbox {dB}$ reduces the rate of the accidental coincidences and the strong suppression of the LP of $(68 \pm 1)$  dB obviates the need for additional bandwidth filtering or background suppression. By adding thin-film elements for band-pass filtering or chromatic pre-compensation, we expect to reduce effects of dispersion and thereby improve the temporal overlap of the pulses. Finally, balancing the loss of both polarization modes will enhance the entanglement further.

To conclude, we demonstrated the hybrid integration of a BRW with the PolyBoard interposer to produce high-quality time-bin entangled photon pairs in the telecom wavelength range. Our results testify to the adequacy of the BRW-PolyBoard PIC for miniaturized quantum communication. We identify the main causes of photon loss and outline a feasible route towards a second generation of significantly enhanced hybrid PICs. Here, the most notable upgrades include transitioning to fiber- or chip-based interferometers, engineering the mode field overlap at the chip interface and employing the already available and greatly improved BRW structures.

### Supplementary Information

Supplementary Information.

## Data Availability

Data underlying the results presented in this paper are available in Ref.^63^.
