# Supplementary material for: Time-bin entanglement at telecom wavelengths from a hybrid photonic integrated circuit
Source: Sci Rep. 2024 May 1;14:9990. doi: 10.1038/s41598-024-60758-4 (PMC11063055; doi:10.1038/s41598-024-60758-4)
Supplement: Supplementary file 1 — Supplementary Information. [file 41598_2024_60758_MOESM1_ESM.pdf]

# Time-bin entanglement at telecom wavelengths from a hybrid photonic integrated circuit: supplemental document

Thiel, H.[1,*], Jehle, L.[2,3], Chapman, R. J.[1,4], Frick, S.[1], Conradi, H.[3], Kleinert, M.[3], Suchomel, H.[5], Kamp, M.[5], Höfling, S.[5], Schneider, C.[5,6], Keil, N[3], and Weihs, G.[1]

[1]Institut für Experimentalphysik, Universität Innsbruck, 6020 Innsbruck, Austria
[2]Faculty of Physics & Vienna Doctoral School in Physics & Vienna Center for Quantum Science and Technology, University of Vienna, 1090 Vienna, Austria
[3]Fraunhofer Institute for Telecommunications, Heinrich-Hertz-Institut, 10587 Berlin, Germany
[4]Optical Nanomaterial Group, Institute for Quantum Electronics, Department of Physics, ETH Zurich, 8093 Zurich, Switzerland
[5]Technische Physik, Universität Würzburg, 97074 Würzburg, Germany
[6]Institute of Physics, University of Oldenburg, 26129 Oldenburg, Germany,
[*]hannah.thiel@uibk.ac.at

February 5, 2024

## 1 PolyBoard

The PolyBoard is fabricated from two different polymers which are iteratively applied via spin-coating on a 4-inch silicon wafer and further processed using photolithography and dry-etching. The waveguides have a quadratic cross-section of $3.2\,\mu$m x $3.2\,\mu$m with an index contrast of $\Delta n = 0.03$ resulting in a simulated mode field that is rotationally symmetric with a $1/e^2$ diameter of $3.9\,\mu$m. The effective index for the transversal-electric (TE) mode is $n_{\text{eff}}^{\text{TE}}$ =1.463 whereas the transversal-magnetic (TM) mode has $n_{\text{eff}}^{\text{TM}}$ =1.462 resulting in a birefringence of $\sim 1 \cdot 10^{-3}$. Using cut-back measurements, we evaluate a propagation loss of $\sim 0.9$ dB/cm for this wafer in separate test structures.

In the presented PolyBoard, thin-film elements (TFE) are used to realize wavelength filtering and polarization splitting which are challenging to implement monolithically as they require a large footprint or exhibit high losses and low extinction ratios. During the fabrication, slots of a few-micrometer thickness are etched into the PolyBoard and are later equipped with the fitting TFE [1]. Because of the small index contrast, the optical loss caused by the unguided propagation through the etched slot is limited and further minimized by appropriate waveguide tapering on each side of the slot. After inserting the TFEs, they are secured with an index-matched and UV-curable adhesive.

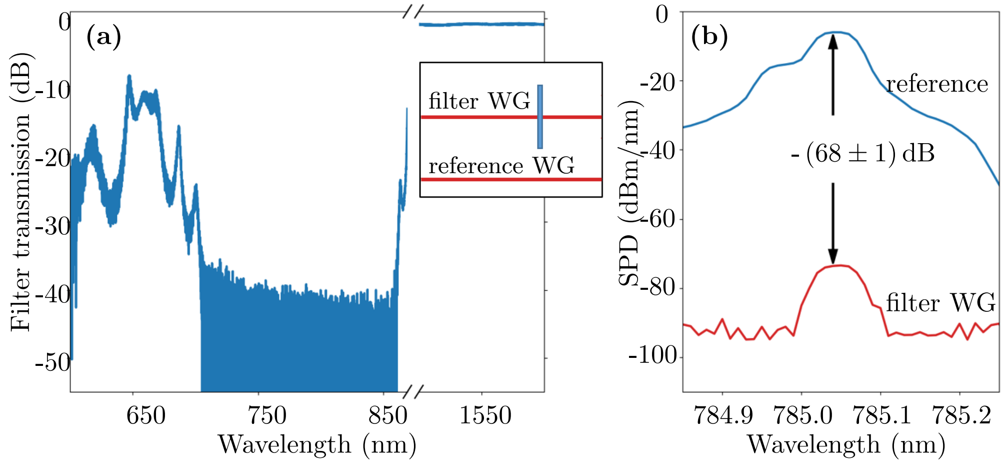


Figure S1: **Transmission measurements for long-pass (LP) filter at test structure.** Filter transmission over a broad spectrum using a white light source and a tunable C-Band laser that is limited by the noise floor of the optical spectrum analyzer for the wavelength range 700 nm to 850 nm (a). To assess the full potential of the LP, a laser diode emitting at 785.05 nm with high spectral power density (*SPD*) was used (b). The filter performance is inferred by subtracting the transmission of the straight reference waveguide (WG) from the transmission of a waveguide passing the filter slot with the LP. The layout of the test structure is shown in the inset of (a) where the red lines indicate the waveguide core and the vertical blue bar the LP.

To assess the performance of the long-pass (LP) filter, we insert it into a test structure depicted in the inset of Fig. S1 (a). By comparing the transmission of a straight reference waveguide and a waveguide passing the LP, we evaluate the suppression. We perform the measurement for three different light sources: a supercontinuum white light laser (*NKT Photonics, SuperK*), a laser diode emitting at 785.05 nm with high spectral power density (*Integrated optics, Matchbox 785nm SLM*), and a tunable laser (*Agilent, 8164B with 81635A and 81689B*) covering the telecom C-band. In this way, we analyze the transmission

for telecom wavelengths, the maximum suppression close to the wavelength used to pump the parametric down-conversion process, and the bandwidth of the filter. We find a suppression of $> 40\,\mathrm{dB}$ for $700\,\mathrm{nm}$ to $850\,\mathrm{nm}$ and a maximum suppression of $(68 \pm 1)\,\mathrm{dB}$ at $785.05\,\mathrm{nm}$.

# 2 Modes and coupling loss

The telecom wavelength optical modes of the Bragg-reflection waveguide (BRW) and the PolyBoard have very different shapes, as shown in Fig. S2. Upon assembly of the photonic integrated circuit, any displacement of the facets with respect to each other leads to a considerable coupling loss, as shown in Fig. S3. For perfect alignment we expect $\sim 2.6\,\mathrm{dB}$ of coupling loss with a horizontal (vertical) tolerance of $\sim 1.1\,\mu\mathrm{m}$ ($\sim 0.7\,\mu\mathrm{m}$) causing additional $1\,\mathrm{dB}$ of loss or $\sim 1.8\,\mu\mathrm{m}$ ($\sim 1.3\,\mu\mathrm{m}$) causing $3\,\mathrm{dB}$.

To ensure optimal coupling, optical feedback is used during the alignment process. We place a lens-capped fiber (SMF28), the BRW, and the preassembled PolyBoard each on a separate multi-axis positioning stage and align them along their common optical axis by eye using a microscope. We prepare a TE-polarized signal close to the degeneracy wavelength ($\approx 1535\,\mathrm{nm}$) and couple it via the lensed fiber to the BRW without physical contact. On the other side, we collect the light in the waveguide of the PolyBoard but leave a small ($\approx 1\,\mu\mathrm{m}$) gap to not scratch the surfaced in the next step. The optical signal is displayed on the computer screen as well as sent directly to the photodetector of the Advanced Positioning Technology (APT) system as feedback. After reaching a sufficiently large signal by manual coupling, the active alignment by closed-loop feedback is enabled for the fiber and PolyBoard stage while the BRWs' position is unchanged. When converged, the piezo controllors are disabled, the Polyboard is brought into contact with the BRW, and the adhesive is applied and UV-cured.

# 3 Interferometers

The three interferometers used for the experiment are actually one physical Michelson interferometer consisting of a 50/50 beam splitter and two corner cube retroreflectors. The pump, signal and idler beams are launched into the interferometer at different heights. All three experience a delay of $3\,\mathrm{ns}$ in the long arm. The output beams are coupled into polarization maintaining fibers. Using a single interferometer for all three beams allows stable operation. However, the beam splitter is not optimized for both wavelength ranges. This compromise contributes to the high losses. To align the three interferometer paths we employ CW lasers (M2 Solstis and Santec TSL-710) and a piezo actuator mounted to one of the retroreflectors. We detect the resulting CW interference fringe using a photo diode (Thorlabs S122C) and optimize the visibilities and transmissivities. This includes balancing the efficiencies in the long and short paths.

For the time-bin measurements we switch to the pulsed Ti:Sapphire laser

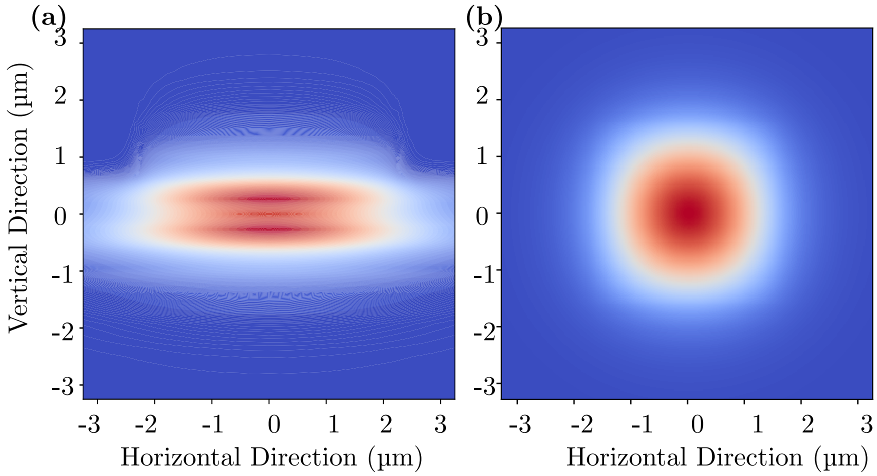


Figure S2: **Simulations of the waveguide modes of BRW and PolyBoard.** The electric field absolute value of the 1550 nm TE-polarized double cigar shaped mode of the BRW (a) and the rotationally symmetric mode of the PolyBoard waveguide (b). The TM-polarized modes are not shown here as they look very similar.

(Coherent Mira 900). To determine the $(91 \pm 5)$ % visibility of the interference in the central time bin, we rotate the glass plate in one of the analysis interferometers and measure the coincidences at every step. To arrive at coincidence counts as a function of phase shift the rotation angle of the glass plate is converted to the path length travelled by the light in the glass plate. We then fit a sine to the measured coincidence data and from this calculate the visibility.

# 4 2D histogram and its interpretation

One sample of the measurements recorded for the time-bin tomography is shown in Fig. S5 (a). Its interpretation is illustrated in Fig. S5 (b). A detection time $t_{-1}$ ($t_{+1}$) represents the single photon state $|1\rangle$ ($|2\rangle$), where a photon measured at either detector took the short (long) path through both the pump and the analysis interferometers. Photons detected at $t_0$ took the short path once and the long path once. The three possible detection times at Alice's and Bob's detectors result in nine positions in the 2D histogram where coincidences can be measured:

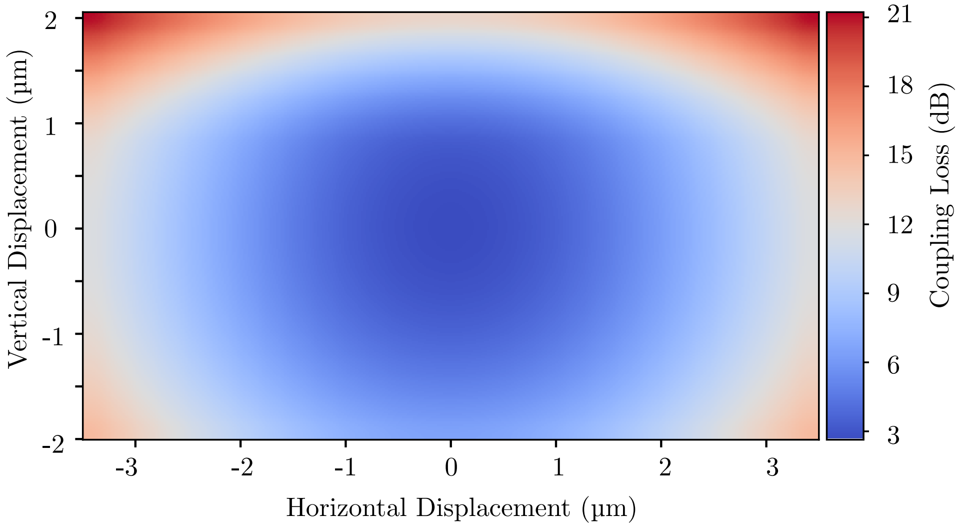


Figure S3: **Coupling loss between BRW and PolyBoard waveguides.** The coupling loss between the PolyBoard mode and BRW mode are calculated using the commercial software Fimmwave. By tuning the relative displacement in both axes in the software, we find a minimum achievable coupling loss of ∼2.6 dB. Due to the layer structure of the BRW, even a small displacement in the vertical direction leads to a significant increase in coupling loss.

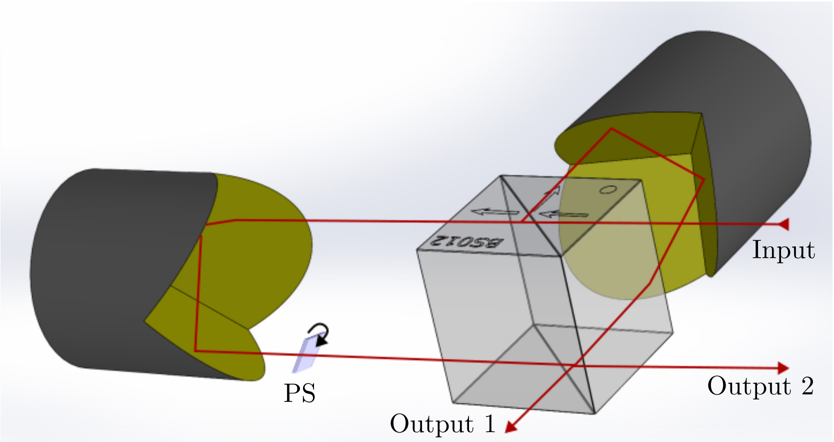


Figure S4: **Free-space interferometer.** The free-space interferometer serves as pump interferometer and analysis interferometers for Alice and Bob. The three beams are launched at different heights, split at a 50/50 beam splitter and reflected by corner cube retroreflectors in the short and long arms. Glass plates in the long arms can be rotated to serve as phase shifters (PS). For clarity the path of only one beam is shown.

| | |
|---|---|
| Position #1 | $\|11\rangle$ |
| Position #2 | $\|+1\rangle$, $\|L1\rangle$ |
| Position #3 | $\|21\rangle$ |
| Position #4 | $\|1+\rangle$, $\|1L\rangle$ |
| Position #5 | $\|++\rangle$, $\|+L\rangle$, $\|L+\rangle$, $\|LL\rangle$ |
| Position #6 | $\|2+\rangle$, $\|2L\rangle$ |
| Position #7 | $\|12\rangle$ |
| Position #8 | $\|+2\rangle$, $\|L2\rangle$ |
| Position #9 | $\|22\rangle$ |

Here $|+\rangle = 1/\sqrt{2}\,(|1\rangle + |2\rangle)$ and $|L\rangle = 1/\sqrt{2}\,(|1\rangle + i\,|2\rangle)$. The states $|12\rangle$ and $|21\rangle$ are not measured if a photon pair is created in either the early or the late pump pulse, as is the case here.

# 5 Density matrix and its eigenvalues

The density matrix $\rho$ reconstructed via maximum likelihood estimation has positive eigenvalues demonstrating that it represents a physical state.

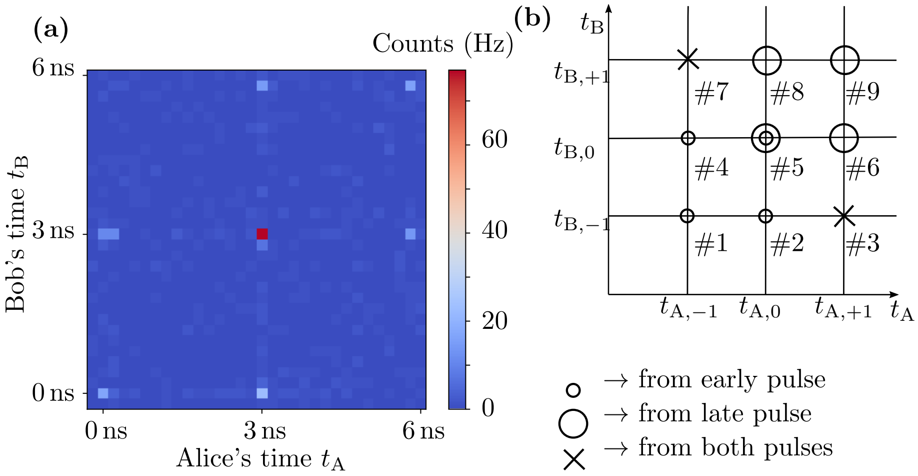


Figure S5: **Triple coincidence between the trigger and Alice's and Bob's detectors.** Measured histogram of the coincidence counts between pump pulse and signal and idler photon arrival times measured for an external pump power of 1mW with 200 ps bin width and 360 s integration time (a). Illustration of the measured data (b). The x-axis (y-axis) corresponds to photon arrival times $t_A$ ($t_B$) at Alice's (Bob's) detector. Possible detection times are $t_{-1}$, $t_0$ and $t_{+1}$ with respect to the pump pulse. The nine positions in the 2D histogram correspond to the different two-photon states.

$$\rho = \begin{pmatrix} 0.4961 + 0.j & 0.1235 + 0.0004j & 0.0211 + 0.0480j & 0.4765 - 0.0703j \\ 0.1235 - 0.0004j & 0.0307 + 0.j & 0.0053 + 0.0119j & 0.1185 - 0.0179j \\ 0.0211 - 0.0480j & 0.0053 - 0.0119j & 0.0055 + 0.j & 0.0135 - 0.0491j \\ 0.4765 + 0.0703j & 0.1185 + 0.0179j & 0.0135 + 0.0491j & 0.4676 + 0.j \end{pmatrix}$$

Eigenvalues = (1.0000+4.4877e-18j, 9.0249e-08+9.0829e-18j, 1.1191e-10-7.4563e-18j, 1.2283e-13+2.7313e-18j)

# References

1. Kleinert, M. et al., Photonic integrated devices and functions on hybrid polymer platform in *Physics and Simulation of Optoelectronic Devices XXV*, **10098** B. Witzigmann, M. Osiński, and Y. Arakawa, International Society for Optics and Photonics (SPIE, 2017), p. 100981A
